# Identification of Tumor-Suppressive *miR-139-3p-*Regulated Genes: *TRIP13* as a Therapeutic Target in Lung Adenocarcinoma

**DOI:** 10.3390/cancers15235571

**Published:** 2023-11-24

**Authors:** Yoko Hagihara, Yuya Tomioka, Takayuki Suetsugu, Masahiro Shinmura, Shunsuke Misono, Yusuke Goto, Naoko Kikkawa, Mayuko Kato, Hiromasa Inoue, Keiko Mizuno, Naohiko Seki

**Affiliations:** 1 Department of Pulmonary Medicine, Graduate School of Medical and Dental Sciences, Kagoshima University, Kagoshima 890-8520, Japan; k5382596@kadai.jp (Y.H.); k4829264@kadai.jp (Y.T.); taka3741@m2.kufm.kagoshima-u.ac.jp (T.S.); k6271399@kadai.jp (M.S.); k8574402@kadai.jp (S.M.); inoue@m2.kufm.kagoshima-u.ac.jp (H.I.); 2 Department of Functional Genomics, Chiba University Graduate School of Medicine, Chuo-ku, Chiba 260-8670, Japan; yusukegoto@chiba-u.jp (Y.G.); naoko-k@hospital.chiba-u.jp (N.K.); mayukokato@chiba-u.jp (M.K.)

**Keywords:** microRNA, lung adenocarcinoma, passenger strand, *miR-139-3p*, *TRIP13*

## Abstract

**Simple Summary:**

Based on the miRNA expression signature of LUAD, we focused on *miR-139-3p*, a passenger strand, and clarified its tumor-suppressive function in lung adenocarcinoma (LUAD) cells. A total of the 21 target genes (*KRT80*, *CENPM*, *SPC24*, *ORC1*, *MYEOV*, *TRIP13*, *GPX8*, *ARHGEF39*, *MKI67*, *KIF18B*, *CHAF1B*, *CP*, *GPRIN1*, *UCK2*, *CHEK1*, *HELLS*, *CTSV*, *FAM111B*, *SLC16A3*, *MELK*, and *CENPF*) were identified as *miR-139-3p* targets in LUAD, and the expression of these genes was as independent prognostic factor for patient survival. Moreover, inhibition of *TRIP13* using a specific inhibitor (DCZ0415) enhanced the sensitivity of LUAD cells to anticancer drugs.

**Abstract:**

Analyses of our microRNA (miRNA) expression signature combined with The Cancer Genome Atlas (TCGA) data revealed that both strands of pre-*miR-139* (*miR-139-5p*, the guide strand, and *miR-139-3p*, the passenger strand) are significantly downregulated in lung adenocarcinoma (LUAD) clinical specimens. Functional analyses of LUAD cells ectopically expressing *miR-139-3p* showed significant suppression of their aggressiveness (e.g., cancer cell proliferation, migration, and invasion). The involvement of the passenger strand, *miR-139-3p*, in LUAD pathogenesis, is an interesting finding contributing to the elucidation of unknown molecular networks in LUAD. Of 1108 genes identified as *miR-139-3p* targets in LUAD cells, 21 were significantly upregulated in LUAD tissues according to TCGA analysis, and their high expression negatively affected the prognosis of LUAD patients. We focused on thyroid hormone receptor interactor 13 (TRIP13) and investigated its cancer-promoting functions in LUAD cells. Luciferase assays showed that *miR-139-3p* directly regulated TRIP13. siRNA-mediated *TRIP13* knockdown and TRIP13 inhibition by a specific inhibitor (DCZ0415) attenuated the malignant transformation of LUAD cells. Interestingly, when used in combination with anticancer drugs (cisplatin and carboplatin), DCZ0415 exerted synergistic effects on cell proliferation suppression. Identifying the molecular pathways regulated by tumor-suppressive miRNAs (including passenger strands) may aid in the discovery of diagnostic markers and therapeutic targets for LUAD.

## 1. Introduction

Lung cancer is the leading cause of cancer-related death worldwide, with approximately 2.3 million new cases of lung cancer and 1.8 million lung cancer-related deaths each year [1]. Patient prognosis is extremely poor, with a 5-year overall survival rate of only 5% for patients with metastatic cancer. Even in patients whose tumors are localized to the lungs, the overall survival rate ranges from 33% to 60% [2]. According to the histological classification of lung cancer, approximately 85% of all lung cancers are non-small-cell lung cancer (NSCLC), the majority of which are lung adenocarcinoma (LUAD) [3]. The treatment methods for LUAD include surgery, radiotherapy, chemotherapy, molecular targeted therapy, and immunotherapy, and the treatment strategy is determined by the disease stage and the presence or absence of driver gene mutations [3]. Molecularly targeted drugs that address genetic mutations in driver genes are being developed rapidly, and many patients will benefit from these drugs [3]. However, in the past, cytotoxic anticancer drugs were the mainstay of drug therapy for advanced-stage LUAD in which molecularly targeted drugs were not applicable. Recently, the effectiveness of immunotherapy has been confirmed, and immunotherapy alone or in combination with anticancer drugs is being used to treat LUAD [4]. It is essential to develop new treatment regimens that increase the effectiveness of anticancer drugs for advanced-stage patients without driver gene mutations.

In the post-genomic era, it has been discovered that the human genome contains an extremely large number of functional RNAs that do not encode proteins. We realized the importance of clarifying their functions in normal and diseased cells [5]. microRNAs (miRNAs) are short (~22 nucleotides) non-coding RNAs that act mainly as gene expression regulators at the post-transcriptional level in a sequence-dependent manner [6]. A single miRNA controls the expression of many different types of genes, and miRNAs play important roles in physiological cellular processes [7].

Numerous studies have shown that the aberrant expression of miRNAs is closely involved in cancer cell malignant transformation, e.g., proliferation, invasion, metastasis, and drug resistance [8]. Traditionally, it was thought that only the guide strand of miRNAs derived from pre-miRNAs was functional in cells, and therefore, cancer research has been focused on the guide strands. Our recent studies revealed that the passenger strand of miRNAs derived from pre-miRNAs is intricately involved in the molecular pathogenesis of human cancers, including lung cancer [9,10]. We can uncover new molecular pathways in cancer cells by examining the molecules regulated by both strands of miRNAs derived from pre-miRNAs.

Recently, we created a miRNA expression signature based on LUAD tissues using RNA sequencing [10]. Analysis of this signature showed that both the guide and passenger strands of pre-*miR-139* (*miR-139-5p* and *miR-139-3p*, respectively) were downregulated in LUAD tissues. Focusing on *miR-139-3p*, we have identified its tumor-suppressive roles and cancer-promoting target genes in various cancer types (e.g., bladder cancer, renal cell carcinoma, oral cancer, and colorectal cancer) [11,12,13,14]. A unique feature of miRNAs is that the target genes of a specific miRNA differ depending on the cell. Our previous studies confirmed that the same tumor-suppressive miRNA regulates different genes depending on the cancer type [11,12,13,14]. The aim of this study was to confirm the tumor-suppressive function of *miR-139-3p* and to clarify the molecular networks controlled by *miR-139-3p* in LUAD cells. Ectopic expression of *miR-139-3p* in cancer cells significantly blocked proliferation and induced cell cycle arrest and apoptosis. Regarding *miR-139-3p* target genes, thyroid hormone receptor interactor 13 (*TRIP13*) was found to be directly regulated by tumor-suppressive *miR-139-3p* in LUAD cells. Aberrant expression of TRIP13 facilitated the malignant transformation of LUAD cells. Importantly, a TRIP13-specific inhibitor (DCZ0415) exerted synergistic effects on the suppression of cell proliferation when used in combination with various anticancer drugs (e.g., cisplatin and carboplatin).

Exploring the oncogenic networks controlled by tumor-suppressive miRNAs, including the passenger strands, will facilitate the identification of therapeutic target molecules for LUAD.

## 2. Materials and Methods

### 2.1. miRNA Expression and Clinical Significance in Patients with LUAD Using an In Silico Database Analysis

We recently created a miRNA expression signature based on LUAD clinical specimens using RNA sequencing [10]. Using this signature, we selected miRNAs with suppressed expression in LUAD tissues. The expression of miRNAs and genes in LUAD clinical tissues was analyzed using the following databases: The Cancer Genome Atlas (TCGA) (https://www.cancer.gov/tcga, accessed on 17 January 2023), FIREBROWSE (http://firebrowse.org/, accessed on 17 January 2023), and Genomic Data Commons Data Portal (https://portal.gdc.cancer.gov/, accessed on 17 January 2023). Overall survival data were obtained from cBioPortal (https://www.cbioportal.org/, accessed on 17 January 2023) and OncoLnc (http://www.oncolnc.org/) (data downloaded on 17 January 2023).

### 2.2. Functional Assay of miRNAs and miRNA Target Genes in LUAD Cells

Two LUAD cell lines, A549 and H1299, were used in the functional assays in this study (American Type Culture Collection, Manassas, VA, USA). Small RNAs (miRNAs and siRNAs) were transfected into LUAD cell lines, and cell proliferation, migration, and invasion were evaluated and compared with those of control cells. The transfection procedures of miRNAs and siRNAs were described in our previous studies [10,15,16,17]. All miRNA precursors were transfected at 10 nM, and all siRNAs were transfected at 5 nM into A549 and H1299 cell lines using RNAiMAX (Invitrogen, Carlsbad, CA, USA). Mock was a group without precursors or siRNAs. Cell cycle analysis was performed using a flow cytometer (BD FACSCelestaTM Flow Cytometer, BD Biosciences, Franklin Lakes, NJ, USA). Details of the cell functional assays are described in our previous paper [10,15,16,17]. The reagents used for these analyses are listed in Table S1.

### 2.3. Identification of Oncogenic Targets Regulated by miR-139-3p in LUAD Cells

To identify putative gene targets controlled by *miR-139-3p* in LUAD, we used TargetScanHuman v8.0 (https://www.targetscan.org/vert_80/, accessed on 24 May 2023) and a gene expression profile from the GEO database (GEO accession number: GSE242241). This expression profile comprises genes whose expression was altered after *miR-139-3p* transfection into A549 cells.

We used GeneCodis4 software to infer the molecular functions of the *miR-139-3p* target genes [18]. Gene set enrichment analysis software was used to infer the molecular pathways controlled by these genes [19,20].

### 2.4. Dual-Luciferase Reporter Assay

We conducted a dual-luciferase reporter assay to confirm that *miR-139-3p* binds directly to the 3′UTR of the *TRIP13* gene. The *miR-139-3p*-binding sequence cloned into the psiCHEK2 vector (C8021; Promega, Madison, WI, USA) is shown in Figure S1. The dual-luciferase reporter assay procedure was described in our previous studies [10,15,16,17]. Transfection of the purified plasmid vectors into LUAD cells was performed using Lipofectamine 2000 (Invitrogen) at 50 ng/well. After 72 h of transfection, we conducted dual-luciferase reporter assays using the Dual Luciferase Reporter Assay System (Promega). The reagents used for these analyses are listed in Table S1.

### 2.5. Anticancer Effects of DCZ0415 in LUAD Cells

To determine the anticancer effects of cisplatin, carboplatin, and DCZ0415 (TRIP13 inhibitor) in LUAD cells, XTT assays were conducted. XTT assays using Cell Proliferation Kits (catalog no.: 20-300-1000, Biological Industries, Beit-Haemek, Israel) were performed to assess cell proliferation. LUAD cells were plated at 4.0 × 104 cells per well in 96-well plates. Then, the half-maximal inhibitory concentration (IC50) values of these drugs were calculated using GraphPad Prism8 software.

### 2.6. Western Blotting and Immunohistochemistry

The procedures for Western blotting and immunohistochemistry have been described in our previous studies [10,15,16,17]. LUAD cell lysates were prepared using a RIPA Lysis Buffer System (catalog no.: sc-24948, Santa Cruz Biotechnology Inc., Dallas, TX, USA). Protein concentrations were measured using a PierceTM BCA Protein Assay Kit (catalog no.: 23227, Thermo Fisher Scientific, Rockford, IL, USA). Protein (20 μg) was injected into each well of SuperSepTM Ace (7.5%, 13 well) (catalog no.: 198-14941, FUJIFILM Wako Pure Chemical Corporation, Osaka, Japan) and electrophoresis was performed. Precision Plus ProteinTM Dual Color Standards (catalog no.: #1610374, Bio-Rad Laboratories, Inc., Hercules, CA, USA) were used as the standard. Proteins were transferred to polyvinylidene fluoride membranes (catalog no.: PPVH00010, Merck KGaA, Darmstadt, Germany). The membranes were incubated with blocking buffer (5% skimmed milk) (catalog no.: 190-12865, FUJIFILM Wako Cells 2023, 12, 1885 4 of 20 Pure Chemical Corporation, Osaka, Japan) in TBST. The signal was detected using Amersham ECL Prime Western Blotting Detection Reagent (Cytiva, Marlborough, MA, USA). The antibodies used in the present study are listed in Table S1.

### 2.7. Statistical Analysis

In this study, statistical analyses were performed using JMP Pro 16 (SAS Institute Inc., Cary, NC, USA). Analysis of differences between the two groups used Welch’s t-test. Differences among multiple groups were analyzed using Dunnett’s test. Patient survival rates were analyzed using Kaplan–Meier survival curves and the log–rank test.

## 3. Results

### 3.1. Tumor-Suppressive Function of miR-139-3p in LUAD Cells

**Figure 1:**
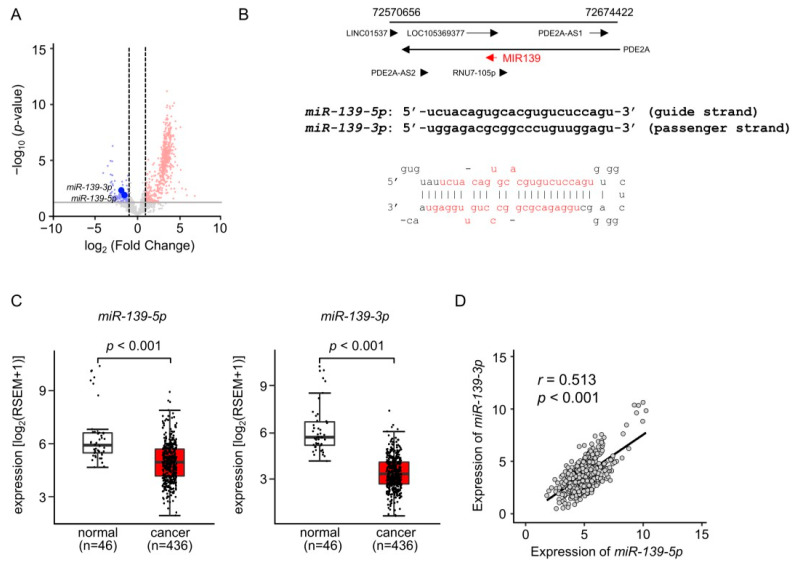
Expression levels of *miR-139-5p* and *miR-139-3p* in LUAD clinical tissues. (**A**) Volcano plot of the miRNA expression signature based on miRNA sequencing (GEO accession number: GSE230229). The log_2_ fold change (FC) in expression is plotted on the *x*-axis and the log_10_ *p*-value on the *y*-axis. The blue and red dots represent the downregulated (log_2_FC < −2.0 and *p* < 0.05) and upregulated (log_2_FC > 2.0 and *p* < 0.05) miRNAs, respectively. (**B**) Chromosomal location of pre-*miR-139* within the human genome. The mature sequences of *miR-139-5p* (guide strand) and *miR-139-3p* (passenger strand) are shown. (**C**) Expression levels of *miR-139-5p* and *miR-139-3p* validated in LUAD clinical specimens. The expression of both miRNAs was significantly downregulated in cancer tissues (*p* < 0.001). (**D**) Positive correlations (Spearman’s rank test) between *miR-139-5p* and *miR-139-3p* expression levels in clinical specimens (*r* = 0.513, *p* < 0.001).

Analysis of our miRNA expression signature created by RNA sequencing showed that both strands of pre-*miR-139* were downregulated in LUAD tissues (Figure 1A). According to the miRNA database miRBase, *miR-139-5p* is annotated as the guide strand derived from pre-*miR-139* and *miR-139-3p* as the passenger strand (Figure 1B). Downregulation of these miRNAs in LUAD clinical specimens was confirmed by analysis of TCGA-LUAD datasets from TCGA (Figure 1C). A positive correlation was detected between *miR-139-5p* and *miR-139-3p* expression levels by Spearman’s rank analysis (*r* = 0.513, *p* < 0.001; Figure 1D).

Our research interests were to elucidate the function of the passenger strands of miRNAs and identify novel oncogenic pathways regulated by tumor-suppressive miRNAs in LUAD cells. The tumor-suppressive roles of *miR-139-3p* were evaluated by transient transfection assays using two LUAD cell lines, A549 and H1299. We also investigated the functional significance of *miR-139-5p* (the guide strand) in LUAD cells. Surprisingly, compared to *miR-139-3p*, *miR-139-5p* had poor tumor suppressive function in LUAD cells (Figure S2).

**Figure 2:**
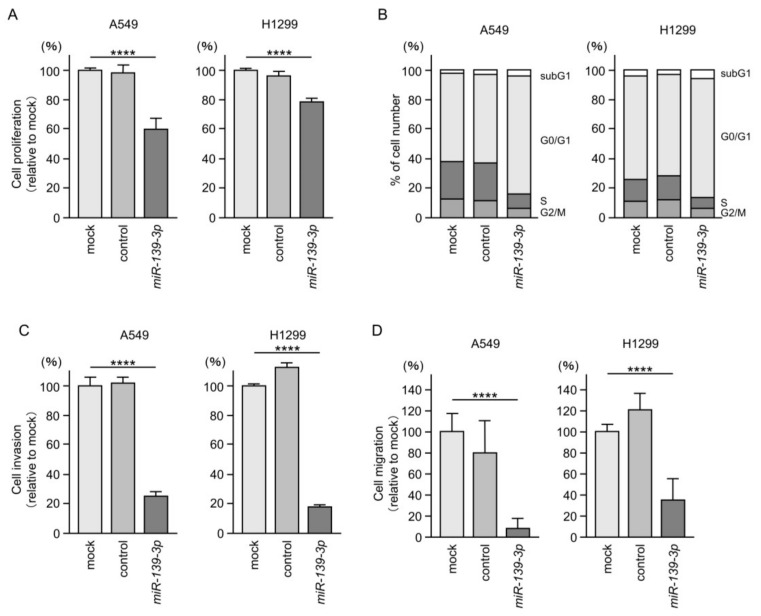
Effects of ectopic expression of *miR-139-3p* in LUAD cells (A549 and H1299). (**A**) Cell proliferation assessed by XTT assay. At 72 h after transient transfection of miRNAs, cancer cell viability was analyzed. (**B**) Cell cycle status at 72 h after transfection with *miR-139-3p* assessed using flow cytometry. (**C**) Cell invasion assessed using Matrigel invasion assays at 48 h after seeding *miR-139-3p*-transfected cells into the chambers. (**D**) Cell migration assessed using a membrane culture system at 48 h after seeding *miR-139-3p* transfected cells into the chambers. ****, *p* < 0.0001.

Cancer cell proliferation was significantly suppressed by ectopic expression of *miR-139-3p* in A549 and H1299 cells (Figure 2A). Cell cycle assays demonstrated an increased proportion of cells in the G0/G1 phase after the induction of *miR-139-3p* expression (Figure 2B). Furthermore, cell invasion and migration were significantly suppressed by ectopic expression of *miR-139-3p* in LUAD cells (Figure 2C,D). Typical images from the invasion and migration assays after *miR-139-3p* transfection are shown in Figure S3.

These results suggest that *miR-139-3p* acts as a tumor-suppressive miRNA in LUAD cells.

### 3.2. Identification of Cancer-Promoting Genes Regulated by miR-139-3p in LUAD Cells

**Figure 3:**
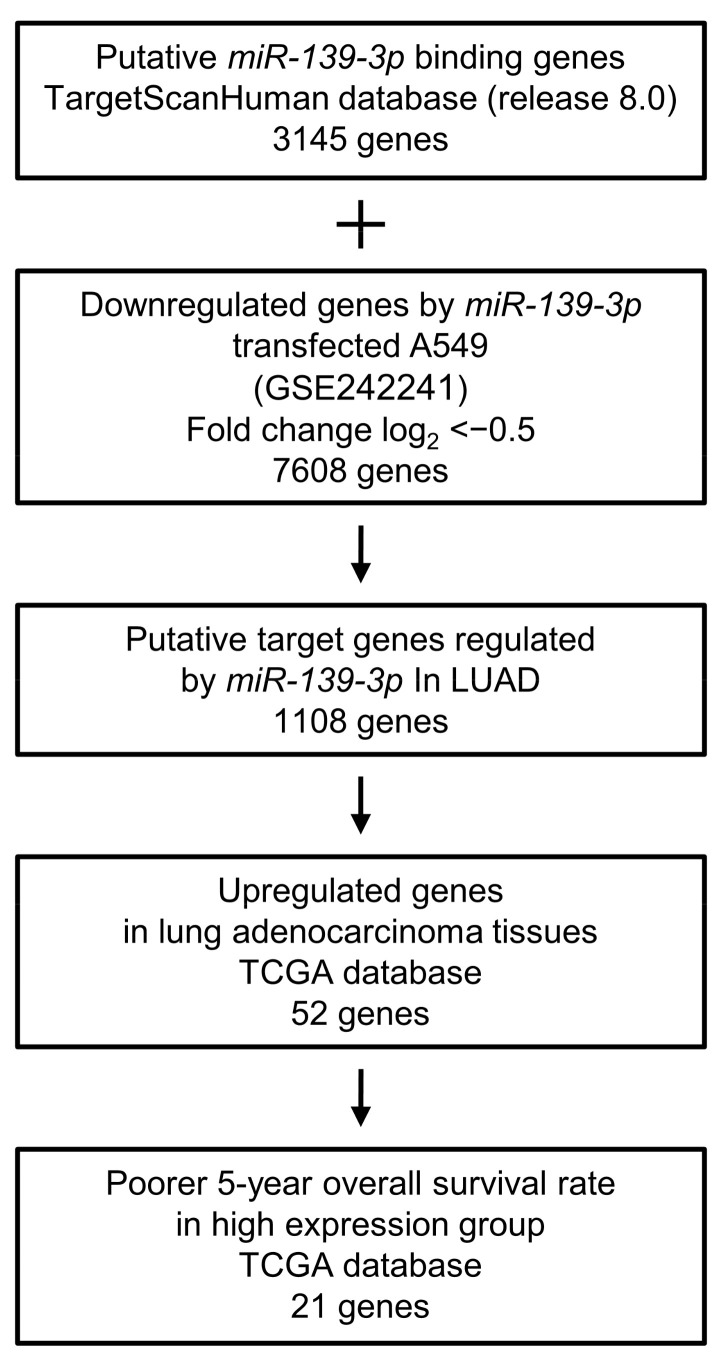
Flowchart for identification of *miR-139-3p* targets in LUAD cell. To identify putative targets of *miR-139-3p* in LUAD cells, the following two datasets were merged: the TargetScanHuman database (release 8.0) and our original mRNA expression profile (*miR-139-3p*-transfected A549 cells; GEO accession number: GSE242241). A total of 1108 genes were identified as putative *miR-139-3p* targets. Furthermore, we searched for genes that were associated with the prognosis of LUAD patients using two databases: GEPIA (http://gepia2.cancer-pku.cn/#analysis (accessed on 17 January 2023)) and OncoLnc (http://www.oncolnc.org (accessed on 17 January 2023)). Of the *miR-139-3p* target genes, 21 were upregulated in LUAD tissues, and these 21 genes were analyzed further.

Next, we aimed to clarify the molecular network regulated by tumor-suppressive *miR-139-3p* in LUAD cells. Our strategy for identifying *miR-139-3p* targets is shown in Figure 3.

In a search of the TargetScanHuman database (release 8.0), 3145 genes were identified as having *miR-139-3p*-binding sequences within their 3′UTR. We applied microarray analysis to search for genes with suppressed expression after *miR-139-3p* transfection in A549 cells. A total of 7608 genes were downregulated by *miR-139-3p* transfection. Gene expression data were deposited in the GEO database (accession number GSE242241). We integrated these two datasets to narrow down the gene list, and 1108 genes remained as candidate *miR-139-3p* targets in LUAD cells.

**Figure 4:**
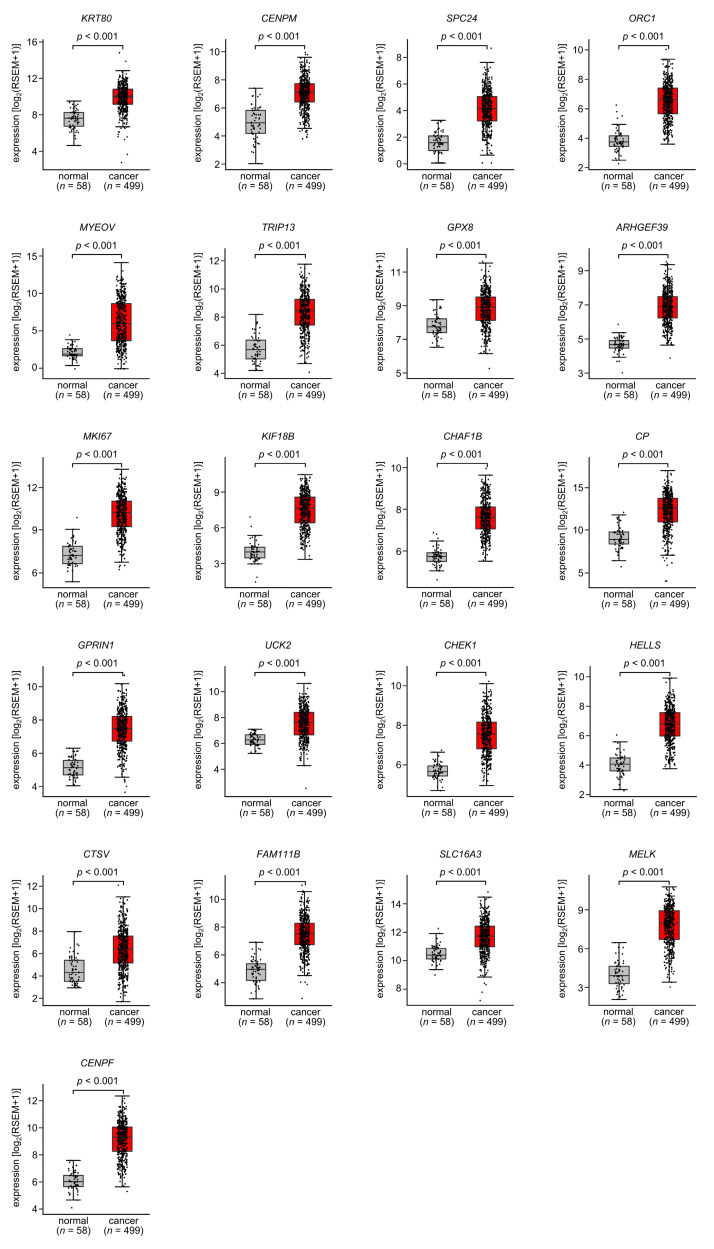
Expression levels of the 21 target genes regulated by miR-139-3p in LUAD. The expression levels of the 21 miR-139-3p target genes (*KRT80*, *CENPM*, *SPC24*, *ORC1*, *MYEOV*, *TRIP13*, *GPX8*, *ARHGEF39*, *MKI67*, *KIF18B*, *CHAF1B*, *CP*, *GPRIN1*, *UCK2*, *CHEK1*, *HELLS*, *CTSV*, *FAM111B*, *SLC16A3*, *MELK*, and *CENPF*) in LUAD clinical specimens were analyzed using the TCGA-LUAD dataset. All genes were upregulated in LUAD tissues (*n* = 499) compared with normal tissues (*n* = 58) (*p* < 0.001).

**Figure 5:**
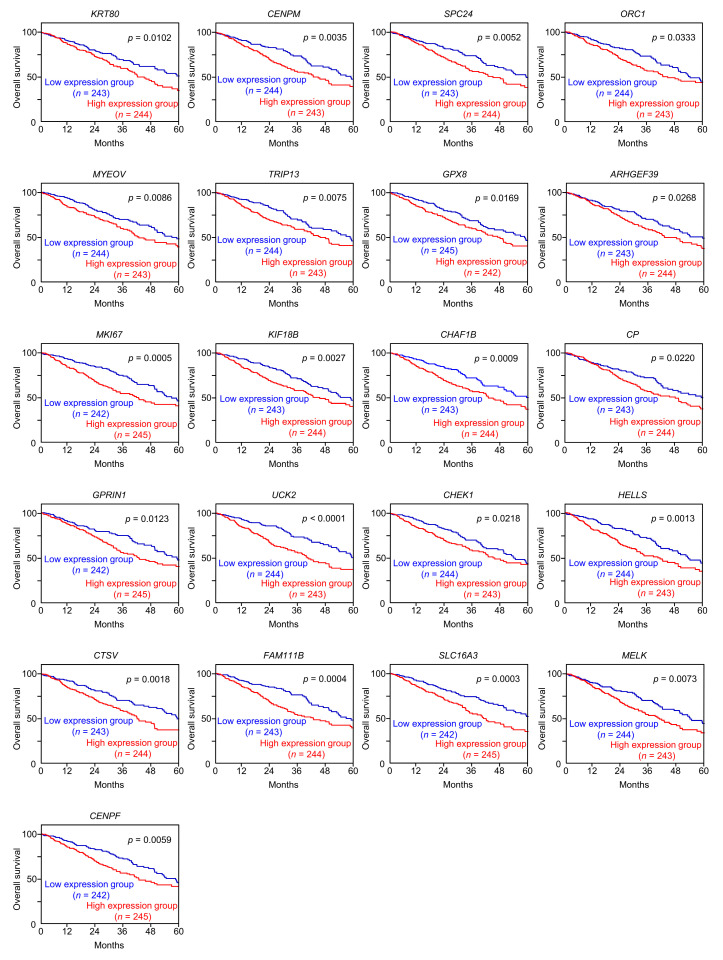
Gene expression in, and 5-year overall survival rate of, patients with LUAD. Kaplan–Meier curves of the 5-year overall survival rates according to the expression of the 21 target genes (*KRT80*, *CENPM*, *SPC24*, *ORC1*, *MYEOV*, *TRIP13*, *GPX8*, *ARHGEF39*, *MKI67*, *KIF18B*, *CHAF1B*, *CP*, *GPRIN1*, *UCK2*, *CHEK1*, *HELLS*, *CTSV*, *FAM111B*, *SLC16A3*, *MELK*, and *CENPF*) are shown. Low expression of all 21 genes was significantly predictive of poorer overall survival in patients with LUAD. The patients (*n* = 487) were divided into high- and low-expression groups according to the median gene expression level. The red and blue lines represent the high and low expression groups, respectively.

Among these target genes, 52 were upregulated in LUAD tissues according to the analysis of the TCGA-LUAD dataset (Table 1), of which 21 genes (Bold) were significantly upregulated (Figure 4). Notably, high expression of these 21 genes was negatively associated with the prognosis of LUAD patients (Figure 5).

Moreover, we investigated the extent to which expression of a negative correlation between *miR-139-3p* and their target genes in LUAD clinical specimens (Figure S4). Spearman’s rank test indicated negative correlations between the expression levels of *miR-139-3p* and 13 target genes (*CENPM*, *SPC24*, *ORC1*, *TRIP13*, *ARHGEF39*, *MKI67*, *KIF18B*, *CHAF1B*, *CHEK1*, *HELLS*, *FAM111B*, *MELK*, and *CENPF*).

### 3.3. Direct Regulation of TRIP13 by miR-139-3p in LUAD Cells

To identify new therapeutic targets for LUAD from among the 52 genes (Table 1), we functionally classified these genes using GeneCodis4 software (https://genecodis.genyo.es/ (accessed on 24 May 2023)) [17]. Among these 52 genes, 13 are involved in “cell cycle” and “DNA replication” (Table 2), and 2 (*TRIP13* and *CENPF*) are involved in cell division checkpoints. Some genes (e.g., *MCM2*, *CHEK1*, *GINS2*, *ADAM8,* and *THY1*) were involved in multiple molecular pathways. Analysis of these genes is a critical issue for understanding the malignant transformation of LUAD. However, genes that did not affect prognosis were excluded from further study. Since an inhibitor is available for TRIP13, we conducted further analyses using the TRIP13 inhibitor DCZ0415.

**Figure 6:**
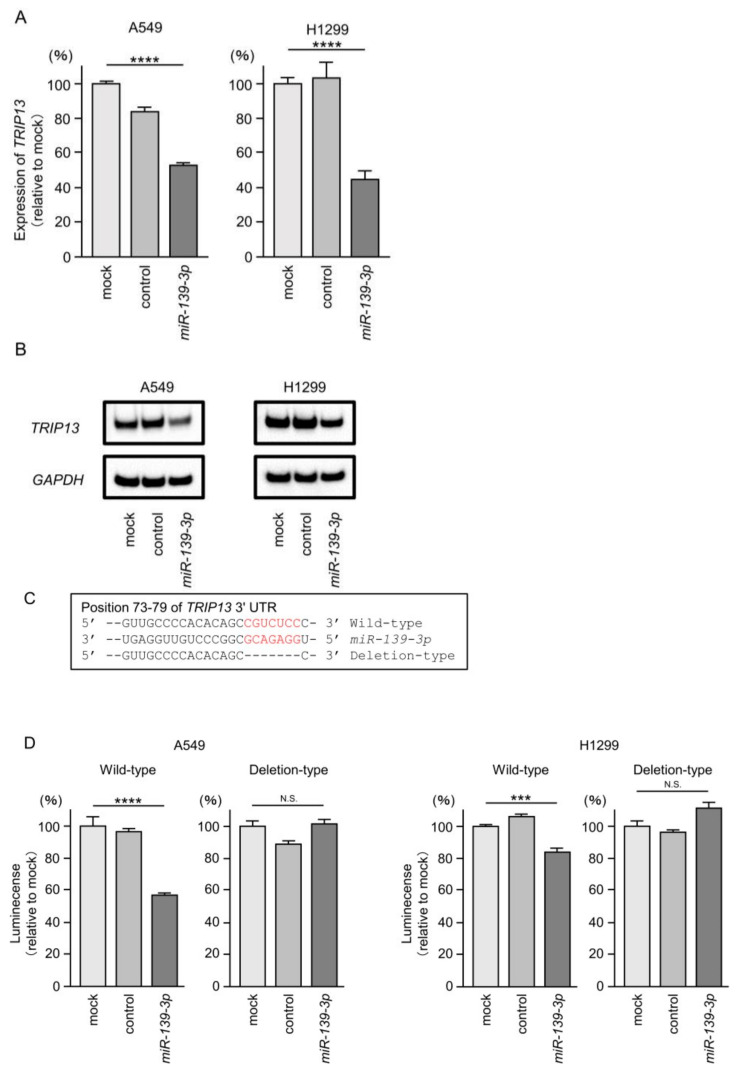
Direct regulation of *TRIP13* by *miR-139-3p* expression in LUAD cells. (**A**) Significant reduction in the *TRIP13* mRNA level by ectopic expression of *miR-139-3p* in LUAD cells (A549 and H1299). Total RNA was extracted 72 h after *miR-139-3p* transfection into LUAD cells, and expression levels were analyzed by real-time PCR. For miRNA expression, *GAPDH* was used as an internal control. (**B**) Significant reduction in the TRIP13 protein level by ectopic expression of *miR-139-3p* in LUAD cells (A549 and H1299). Protein level expression was determined by Western blotting. Proteins were collected 72 h after *miR-139-3p* transfection. GAPDH was used as an internal control. (**C**) Putative *miR-139-3p* binding sites in the 3′UTR of the *TRIP13* gene detected in the TargetScanHuman database (release 8.0). (**D**) Direct binding of *miR-139-3p* to target sequences was analyzed by dual luciferase reporter assays. These data showed that *miR-139-3p* bound directly to the target sequence. ***, *p* < 0.001; ****, *p* < 0.0001; N.S., not significant.

First, we confirmed that TRIP13 mRNA and protein levels were suppressed by ectopic expression of *miR-139-3p* in LUAD cells (Figure 6A,B). Full-size images of the Western blots are shown in Figure S5.

Next, we confirmed by luciferase reporter assay that *miR-139-3p* binds directly to the 3’UTR of the *TRIP13* gene. The putative *miR-139-3p*-binding sequence within *TRIP13* is shown in Figure 6C. Luciferase activity was significantly suppressed when *miR-139-3p* and a vector containing a *miR-139-3p*-binding sequence were simultaneously transfected into LUAD cells (Figure 6D). In contrast, when a vector lacking the *miR-139-3p*-binding sequence was used, no reduction in luciferase activity was observed (Figure 6D).

These results revealed that *miR-139-3p* binds directly to the predicted binding sites in *TRIP13* genes and regulates its expression in LUAD cells.

### 3.4. Expression and Clinical Significance of TRIP13 in LUAD

**Figure 7:**
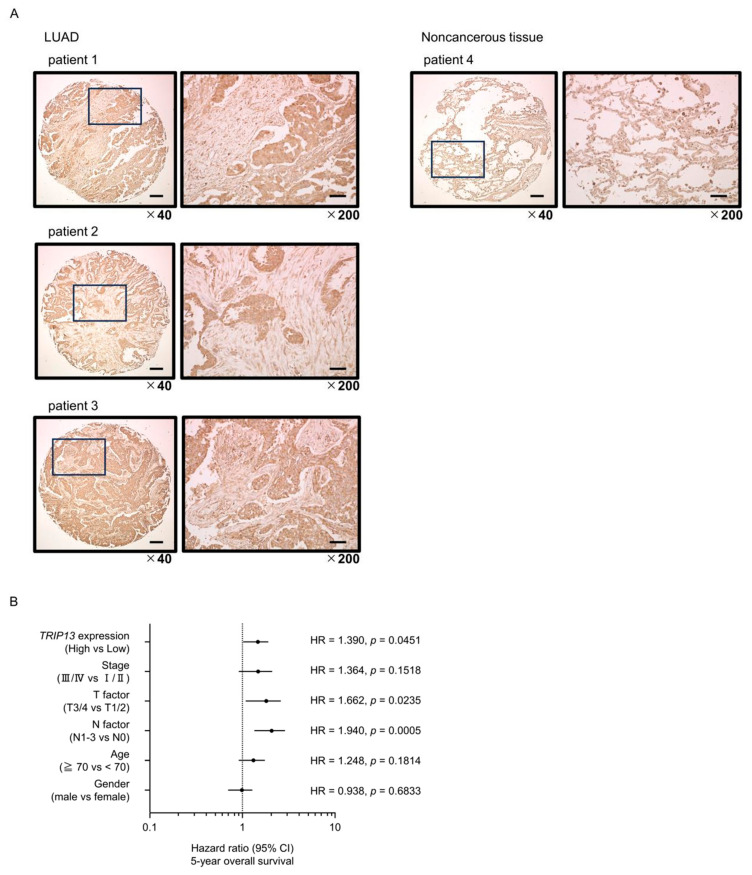

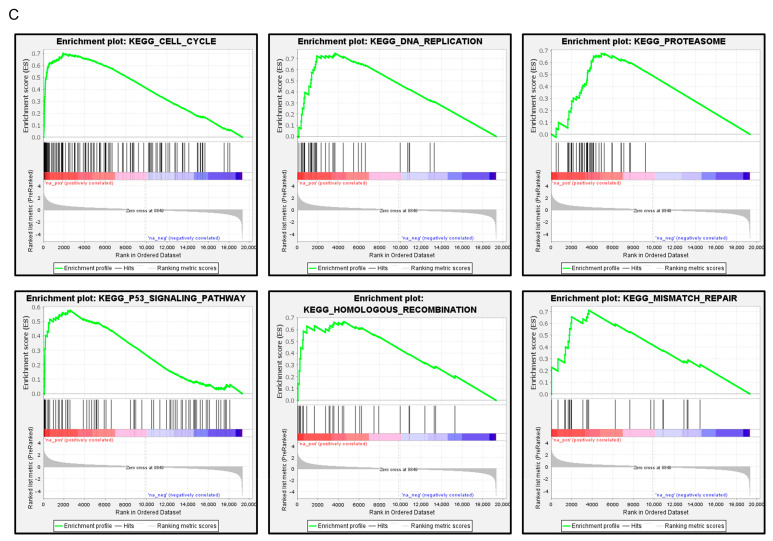
Clinical significance of *TRIP13* expression in LUAD. (**A**) Immunohistochemical staining of TRIP13. Immunostaining showed that the TRIP13 protein was strongly expressed in cancer lesions and less in non-cancerous tissues. Scale bar: 200 µm (low magnification); 50 µm (high magnification). (**B**) Forest plot showing the results of multivariate Cox proportional hazards regression analysis of the 5-year overall survival rate. Patients with high *TRIP13* expression had a significantly lower overall survival rate. These data were obtained from TCGA-LUAD datasets. (**C**) Gene set enrichment analysis (GSEA) was applied to explore molecular pathways mediated by *TRIP13* in LUAD cells. The top six pathways enriched in LUAD patients with high *TRIP13* expression were cell cycle, DNA replication, proteasome, P53 signaling pathway, homologous recombination, and mismatch repair.

Immunostaining was performed to verify the localization of TRIP13 expression in LUAD clinical specimens. Stronger immunostaining of the TRIP13 protein was observed in cancer than in normal lung tissues (Figure 7A).

Multivariate analysis identified *TRIP13* expression as an independent prognostic factor for LUAD after adjusting for the clinical prognostic factors of Stage, T-factor, N-factor, age, and gender (Figure 7B). Specifically, high *TRIP13* expression was associated with a poorer 5-year overall survival rate.

Furthermore, we performed gene set enrichment analysis using TCGA-LUAD data to investigate which molecular pathways were dysregulated in LUAD patients with high *TRIP13* expression. The “cell cycle”, “DNA repair”, “proteasome”, “P53 signaling pathway”, homologous recombination”, and “mismatch repair” pathways were found to be enriched in patients with high *TRIP13* expression (Table 3, Figure 7C). It has been revealed that high expression of *TRIP13* affected the prognosis of LUAD patients. An important question is which molecular pathway is affected by the high expression of the *TRIP13* gene in lung cancer cells.

### 3.5. Effects of TRIP13 Knockdown by siRNAs and TRIP13 Inhibition by a Specific Inhibitor in LUAD Cells

**Figure 8:**
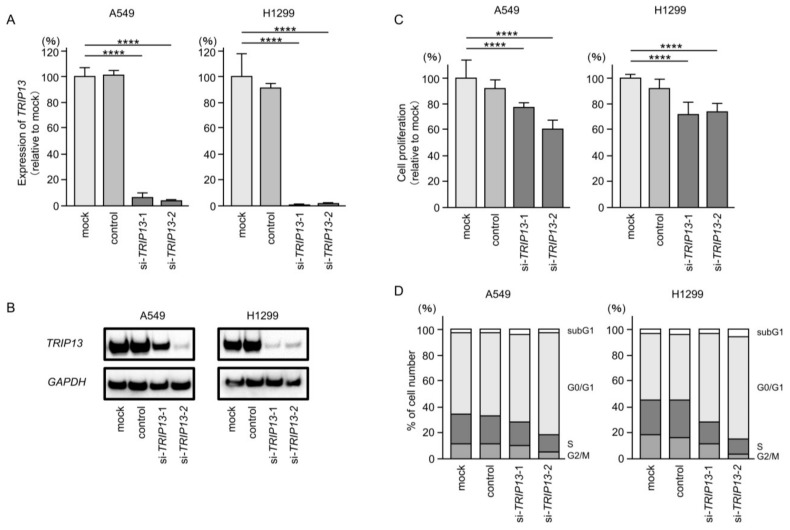
Effects of knockdown of *TRIP13* by siRNAs in LUAD cells (A549 and H1299). (**A**) *TRIP13* mRNA levels were effectively blocked by each siRNA in LUAD cells (A549 and H1299). (**B**) *TRIP13* protein levels were effectively inhibited by two siRNAs (si*TRIP13*-1 and si*TRIP13*-2) in LUAD cells (A549 and H1299). (**C**) Cell proliferation was assessed using XTT assays 72 h after siRNA transfection into LUAD cells. Cell proliferation was significantly blocked after transient transfection of siRNAs. (**D**) Flow cytometry analysis of cell cycle status 72 h after transfection with si*TRIP13*-1 and si*TRIP13*-2. ****, *p* < 0.0001.

To investigate the functional significance of *TRIP13* in LUAD cells, we performed analysis using siRNA to knock down *TRIP13*. The two siRNAs used in this study (si*TRIP13*-1 and si*TRIP13*-2) were confirmed to significantly suppress *TRIP13* expression (both at the mRNA and protein levels) in LUAD cells (Figure 8A,B). Full-size images of the Western blots are shown in Figure S6.

Cell proliferation was significantly suppressed after transfection of both siRNAs in the LUAD cells (Figure 8C). Cell cycle assays demonstrated an increased proportion of cells in the G0/G1 phase after transfection of both siRNAs in LUAD cells (Figure 8D).

### 3.6. Combined Effects of a TRIP13 Inhibitor (DCZ0415) and Anticancer Drugs on LUAD Cells

**Figure 9:**
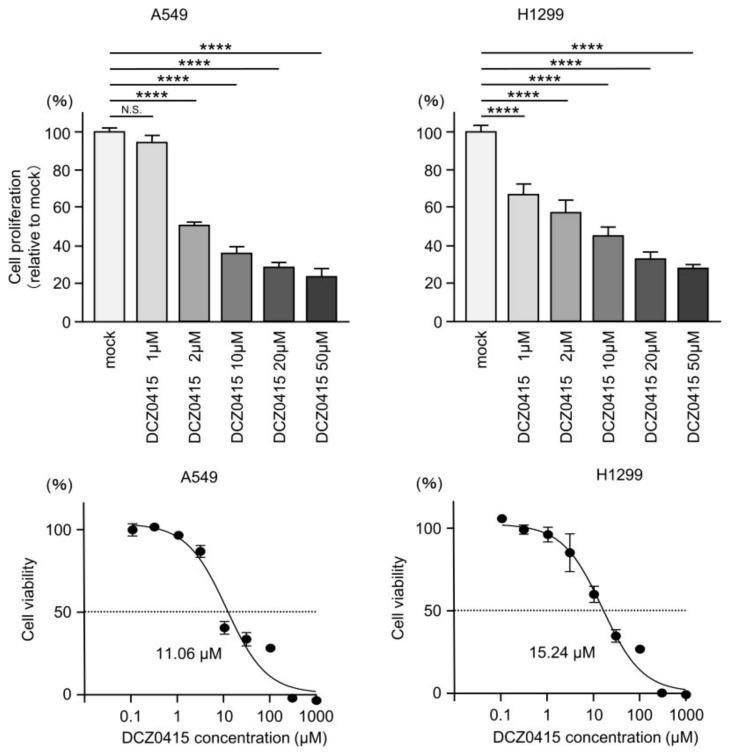
Effects of treatment with DCZ0415 (TRIP13 inhibitor) on LUAD cells (A549 and H1299). The proliferation of LUAD cells was significantly inhibited by DCZ0415 in a concentration-dependent manner. ****, *p* < 0.0001; N.S., not significant.

We also investigated the anticancer effects of a TRIP13 inhibitor (DCZ0415) combined with anticancer drugs (cisplatin and carboplatin) on LUAD cells. We found that DCZ0415 inhibited the proliferation of LUAD cells in a concentration-dependent manner (Figure 9).

**Figure 10:**
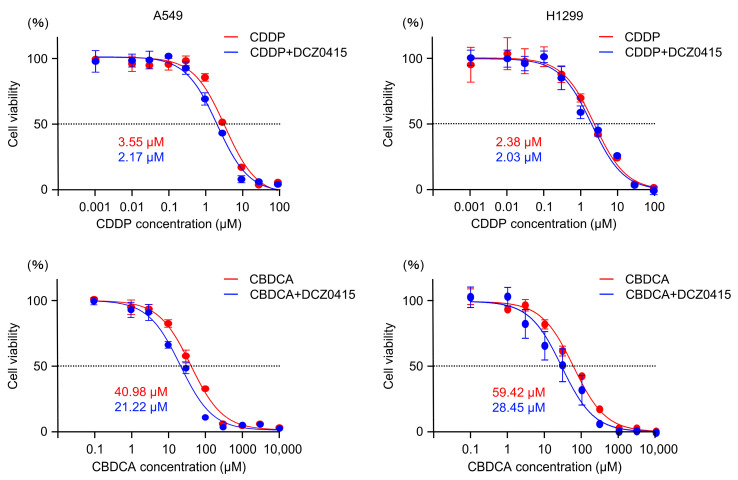
Effects of co-treatment of DCZ0415 (TRIP13 inhibitor) with anticancer drugs (cisplatin and carboplatin) in LUAD cells (A549 and H1299). LUAD cells showed increased sensitivity to anticancer drugs (cisplatin and carboplatin) when co-treated with DCZ0415. CDDP, cisplatin; CBDCA, carboplatin.

Furthermore, the sensitivity of LUAD cells to anticancer drugs (cisplatin and carboplatin) was increased by DCZ0415. Co-treatment of DCZ0415 together with cisplatin or carboplatin resulted in increased sensitivity of A549 and H1299 cells to cisplatin or carboplatin (Figure 10). The IC50 of cisplatin significantly decreased from 3.55 µM to 2.17 µM in A549 cells and from 2.38 µM to 2.03 µM in H1299 cells. The IC50 of carboplatin significantly decreased from 40.98 µM to 21.22 µM in A549 cells and from 59.42 µM to 28.45 µM in H1299 cells.

**Figure 11:**
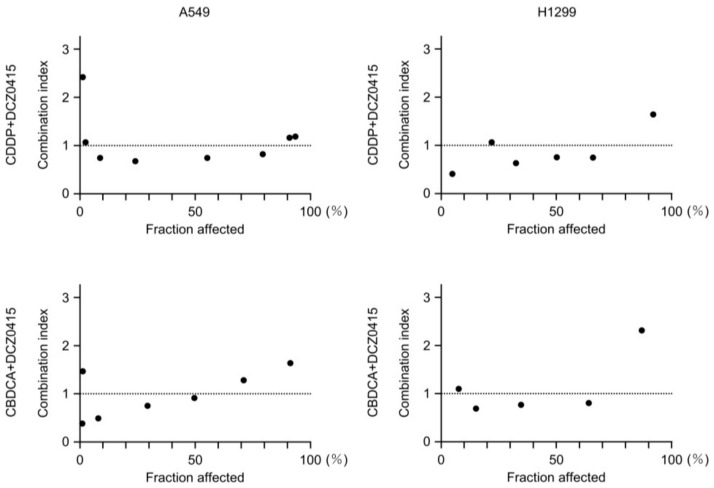
Synergistic effects between two anticancer drugs (cisplatin and carboplatin) and the TRIP13 inhibitor DCZ0415. The Chou–Talalay method was used to determine the synergistic effects between two anticancer drugs (cisplatin and carboplatin) and DCZ0415 in LUAD cells (A549 and H1299). CDDP, cisplatin; CBDCA, carboplatin.

Additionally, the Chou–Talalay method was used to determine the synergistic effect of two anticancer drugs (cisplatin and carboplatin) and DCZ0415. The results revealed that simultaneous administration of anticancer drugs and DCZ0415 had synergistic effects (Figure 11).

## 4. Discussion

In miRNA biogenesis, miRNAs, which function as single-stranded RNAs, are derived from miRNA precursors. Of the two single strands derived from a miRNA precursor, the functional strand is defined as the guide strand and the non-functional strand as the passenger strand [21,22]. Generally, the miRNAs evaluated in cancer research have been guide strands.

The precursor-*miR-139* we focused on in this study is located within the second intron of the phosphodiesterase 2A (PDE2A) gene on chromosome 11q13.4 [23]. The guide strand derived from precursor-*miR-139* is *miR-139-5p*, on which many studies have been conducted [23,24]. In cancer research, the downregulation of *miR-139-5p* has been reported in most cancer types (e.g., breast, gastric, colorectal, bladder, and head and neck cancers, among others) [25,26,27,28,29]. In contrast, a high expression of *miR-139-5p* has been reported in cancer tissues compared with normal tissues in gastrointestinal stromal tumors [23,30]. In lung cancer, several studies showed that *miR-139-5p* was downregulated in LUAD tissues and that it functions as a tumor suppressor by targeting several cancer-related genes [23,31,32,33,34].

From previous reports, it is clear that aberrant expression of *miR-139-5p* plays important roles in various types of cancer cells. In contrast, there have not been many studies on the role of *miR-139-3p*, the passenger strand, in cancer cells.

We have generated miRNA expression signatures for various cancer types using RNA sequencing and selected tumor-suppressive miRNA candidates based on those signatures [10,35,36,37]. Of these candidates, we identified downregulation and tumor-suppressive functions of *miR-139-3p* in various cancers, including bladder cancer, renal cell carcinoma, head and neck squamous cell carcinoma, and colorectal cancer [11,12,13,14]. Here, we showed that *miR-139-3p* functions as a tumor-suppressive miRNA in LUAD in addition to other cancer types.

Previous studies showed that *miR-139-3p* is downregulated in NSCLC tissues and that its expression suppresses cancer cell aggressiveness in vitro and in vivo [38,39]. Furthermore, *ELAVL1* is directly regulated by *miR-139-3p* in NSCLC cells [38]. *ELAVL1* is a member of the ELAVL family of RNA-binding proteins, which contain several RNA recognition motifs, and *ELAVL1* overexpression has been observed in several cancers [40,41,42,43,44]. More recently, downregulation of *miR-139-3p* was detected in lung squamous cell carcinoma, and expression of *miR-139-3p* restrained cell growth and accelerated the cell cycle in this cancer [45]. Moreover, the expression of checkpoint kinase 1 (*CHEK1*) was regulated directly by *miR-139-3p* in lung squamous cell carcinoma cells [45]. CHEK1 is a serine/threonine protein kinase and a key modulator of DNA damage checkpoints [46,47]. Our present analysis and previous reports revealed that *miR-139-3p* has tumor-suppressive functions in lung cancer.

Next, we aimed to elucidate the oncogenic network that is controlled by tumor-suppressive *miR-139-3p* in LUAD cells. In this study, 52 genes were identified as putative targets of *miR-139-3p* in LUAD cells. Among these target genes, 21 were closely involved in the molecular pathogenesis of LUAD. Functional classification of these genes revealed that 11 (*HELLS*, *MELK*, *CHEK1*, *CHAF1B*, *CENPF*, *KIF18B*, *MKI67*, *SPC24*, *FAM111B*, *ORC1*, and *TRIP13*) are involved in the cell cycle, DNA replication, mitotic checkpoint, and cell division. A very interesting finding is that *miR139-3p*, a passenger strand, controls genes essential for cell division and maintenance.

The AAA (ATPases associated with various cellular activities) protein family is widely conserved from bacteria to humans, and these ATPases use the energy from ATP hydrolysis to unfold proteins and dissociate complexes [48,49]. TRIP13 is a member of the AAA protein family, and it functions as a spindle assembly checkpoint for accurate chromosome segregation during cell division [50]. A recent report showed that TRIP13 plays an important role as a gatekeeper for cell maintenance and management (e.g., DNA break repair, recombination, and chromosome synapsis) [51,52].

Aberrant expression of TRIP13 has been reported in a wide range of cancers, including lung cancer [50,53,54]. The knockdown of *TRIP13* in cancer cells significantly suppressed the malignant phenotypes of the cancer cells (i.e., cell proliferation, invasion, and drug resistance) [55,56]. In bladder cancer, overexpression of *TRIP13* enhanced the resistance of cancer cells to cisplatin and doxorubicin [56]. In head and neck cancer, epidermal growth factor receptor (EGFR)-mediated phosphorylation of TRIP13 (pY56) enhanced non-homologous end-joining repair and induced radiation resistance in cancer cells [57]. More recently, overexpression of TRIP13 promoted gefitinib (EGFR inhibitor) resistance in NSCLC cells by controlling autophagy and activating EGFR-mediated oncogenic signaling pathways [58]. Since TRIP13 overexpression is involved in drug resistance in various cancer cells, TRIP13 might be a potential target molecule for cancer therapy. TRIP13 overexpression is common in BRCA1-deficient cancers, confers PARP inhibitor resistance, and correlates with poor prognosis [59].

Recent studies have reported that various noncoding RNA molecules are involved in the aberrant expression of *TRIP13* in cancer cells. *miR-515-5p* was found to be downregulated in prostate cancer tissues, and *TRIP13* was regulated directly by *miR-515-5p* [60]. In hepatocellular carcinoma (HCC), upregulated *TRIP13* induced malignant transformation of HCC in vitro and lung metastasis in vivo [61,62]. In addition, *miR-192-5p* regulated *TRIP13* expression in HCC cells [63]. In colorectal cancer, aberrant expression of *TRIP13* contributed markedly to the aggressive phenotype of cancer cells, and its overexpression induced the downregulation of *miR-129-5p* and *miR-4693-5p* [64,65]. More recently, *miR-30c-1-3p* and *miR-30c-2-3p* were found to be significantly downregulated in breast cancer (BrCa) tissues, and their target gene TRIP13 was overexpressed in BrCa tissues [55]. Moreover, overexpression of *TRIP13* facilitated the aggressiveness of BrCa cells [55]. Based on this study and previous data, *TRIP13* overexpression is partially due to the downregulation of several tumor-suppressive miRNAs in cancer cells.

## 5. Conclusions

Based on the miRNA expression signature of LUAD, we focused on *miR-139-3p*, a passenger strand, and clarified its tumor-suppressive function in LUAD cells. We revealed that *miR-139-3p* target molecules are intricately involved in the molecular pathogenesis of LUAD. Inhibition of *TRIP13* using a specific inhibitor (DCZ0415) attenuated the malignant transformation of LUAD cells. Interestingly, DCZ0415 exhibited a synergistic effect on the suppression of cell proliferation when used in combination with anticancer drugs. TRIP13 is a potential therapeutic target for LUAD.

## Figures and Tables

**Table 1:** Putative target genes regulated by *miR-139-3p* in A549 cells.

Gene ID	Gene Symbol	Gene Name	*miR-139-3p* Total Sites	*miR-139-3p* Transfectant Log_2_ FC
8038	*ADAM12*	ADAM metallopeptidase domain 12	1	−4.61
3898	*LAD1*	ladinin 1	1	−3.28
144501	***KRT80***	keratin 80	1	−2.86
4171	*MCM2*	minichromosome maintenance complex component 2	1	−2.84
79019	***CENPM***	centromere protein M	1	−2.78
147841	***SPC24***	SPC24, NDC80 kinetochore complex component	1	−2.70
4998	***ORC1***	origin recognition complex, subunit 1	1	−2.70
26579	***MYEOV***	myeloma overexpressed	1	−2.57
201266	*SLC39A11*	solute carrier family 39, member 11	1	−2.56
9319	***TRIP13***	thyroid hormone receptor interactor 13	1	−2.53
493869	***GPX8***	glutathione peroxidase 8 (putative)	2	−2.42
84904	***ARHGEF39***	Rho guanine nucleotide exchange factor (GEF) 39	2	−2.35
1734	*DIO2*	deiodinase, iodothyronine, type II	1	−2.27
84733	*CBX2*	chromobox homolog 2	1	−2.24
4288	***MKI67***	antigen identified by monoclonal antibody Ki-67	1	−2.18
51659	*GINS2*	GINS complex subunit 2 (Psf2 homolog)	1	−2.14
116372	*LYPD1*	LY6/PLAUR domain containing 1	1	−2.10
202915	*TMEM184A*	transmembrane protein 184A	1	−2.10
146909	***KIF18B***	kinesin family member 18B	1	−1.96
26256	*CABYR*	calcium-binding tyrosine-(Y)-phosphorylation regulated	1	−1.96
8270	*LAGE3*	L antigen family, member 3	1	−1.89
25837	*RAB26*	RAB26, member RAS oncogene family	1	−1.82
8645	*KCNK5*	potassium channel, subfamily K, member 5	3	−1.79
8208	***CHAF1B***	chromatin assembly factor 1, subunit B (p60)	1	−1.78
1763	*DNA2*	DNA replication helicase/nuclease 2	1	−1.70
4234	*METTL1*	methyltransferase like 1	1	−1.65
1356	***CP***	ceruloplasmin (ferroxidase)	1	−1.61
51237	*MZB1*	marginal zone B and B1 cell-specific protein	1	−1.57
6723	*SRM*	spermidine synthase	1	−1.52
51629	*SLC25A39*	solute carrier family 25, member 39	1	−1.46
114787	***GPRIN1***	G protein regulated inducer of neurite outgrowth 1	1	−1.42
27286	*SRPX2*	sushi-repeat containing protein, X-linked 2	1	−1.41
55612	*FERMT1*	fermitin family member 1	1	−1.22
7371	***UCK2***	uridine-cytidine kinase 2	3	−1.21
1111	***CHEK1***	nudix hydrolase 21	1	−1.19
154467	*CCDC167*	coiled-coil domain containing 167	1	−1.18
3070	***HELLS***	helicase, lymphoid-specific	1	−1.15
1515	***CTSV***	cathepsin V	1	−1.14
101	*ADAM8*	ADAM metallopeptidase domain 8	1	−1.13
374393	***FAM111B***	family with sequence similarity 111, member B	1	−1.10
7070	*THY1*	Thy-1 cell surface antigen	1	−1.08
128272	*ARHGEF19*	Rho guanine nucleotide exchange factor (GEF) 19	1	−1.03
9123	***SLC16A3***	solute carrier family 16 (monocarboxylate transporter), member 3	1	−1.02
65268	*WNK2*	WNK lysine deficient protein kinase 2	1	−0.97
5163	*PDK1*	pyruvate dehydrogenase kinase, isozyme 1	4	−0.96
9833	***MELK***	maternal embryonic leucine zipper kinase	1	−0.75
1063	***CENPF***	centromere protein F, 350/400 kDa	1	−0.72
6659	*SOX4*	SRY (sex determining region Y)-box 4	1	−0.67
146857	*SLFN13*	schlafen family member 13	1	−0.63
51087	*YBX2*	Y box binding protein 2	2	−0.62
55502	*HES6*	hairy and enhancer of split 6 (Drosophila)	1	−0.60
51114	*ZDHHC9*	zinc finger, DHHC-type containing 9	1	−0.57

**Table 2:** Significantly enriched annotations of target genes regulated by *miR-139-3p*.

Description	*p*-Value	FDR	Genes
DNA replication	<0.001	<0.001	*MCM2*, *CHEK1*, *CHAF1B*, *FAM111B*, *GINS2*, *ORC1*, *DNA2*
cell cycle	<0.001	0.006	*HELLS*, *MELK*, *MCM2*, *CHEK1*, *CHAF1B*, *CENPF*, *KIF18B*, *MKI67*, *SPC24*
positive regulation of cellular extravasation	<0.001	0.013	*ADAM8*, *THY1*
integrin-mediated signaling pathway	<0.001	0.013	*ADAM12*, *FERMT1*, *ADAM8*, *THY1*
double-strand break repair via break-induced rep- lication	<0.001	0.027	*MCM2*, *GINS2*
intrinsic apoptotic signaling pathway in response to oxidative stress	0.001	0.041	*MELK*, *PDK1*
negative regulation of the timing of anagen	0.003	0.050	*FERMT1*
DNA replication, Okazaki fragment processing	0.003	0.050	*DNA2*
mitotic spindle assembly checkpoint signaling	0.002	0.050	*CENPF*, *TRIP13*
DNA unwinding involved in DNA replication	0.001	0.050	*MCM2*, *GINS2*
negative regulation of neuron projection regener- ation	0.003	0.050	*THY1*
positive regulation of fibronectin-dependent thy- mocyte migration	0.003	0.050	*ADAM8*
regulation of transcription from RNA polymerase II promoter in response to UV-induced DNA damage	0.003	0.050	*CHEK1*
positive regulation of N-terminal peptidyl-lysine acetylation	0.003	0.050	*SOX4*
DNA replication initiation	0.002	0.050	*MCM2*, *ORC1*
development of primary sexual characteristics	0.003	0.050	*CBX2*
exocrine system development	0.003	0.050	*RAB26*

FDR, false discovery rate.

**Table 3:** *TRIP13*-mediated pathways by Gene Set Enrichment Analysis (GSEA).

Pathway	Enrichment Score	Normalized Enrichment Score	*p*-Value	FDR
KEGG_CELL_CYCLE	0.70	2.60	<0.001	<0.001
KEGG_DNA_REPLICATION	0.75	2.26	<0.001	<0.001
KEGG_PROTEASOME	0.68	2.08	<0.001	<0.001
KEGG_P53_SIGNALING_PATHWAY	0.58	1.96	<0.001	0.002
KEGG_HOMOLOGOUS_RECOMBINATION	0.67	1.93	<0.001	0.002
KEGG_MISMATCH_REPAIR	0.71	1.92	<0.001	0.002

FDR, false discovery rate.

## Data Availability

Publicly available datasets were analyzed in this study. These data can be accessed here: https://www.ncbi.nlm.nih.gov/geo/query/acc.cgi?acc=GSE230229 (accessed on 19 September 2023) and https://www.ncbi.nlm.nih.gov/geo/query/acc.cgi?acc=GSE242241 (accessed on 19 September 2023).

## Supplementary Materials

The following supporting information can be downloaded at: https://www.mdpi.com/article/10.3390/cancers15235571/s1, Figure S1: The vector insertion sequence by luciferase-reporter assay; Figure S2: Effects of ectopic expression of *miR-139-5p* and *miR-139-3p* in LUAD cells (A549 and H1299). *, *p* < 0.05; ****, *p* < 0.0001; N.S., not significant; Figure S3: Atipical images of cell migration or cell invasion assays; Figure S4: Expression correlation between *miR-139-3p* and their target genes in LUAD clinical specimens; Figure S5: full-sized images of Western blot analysis (*TRIP13* antibody) following ectopic expressions of *miR-139-3p* in A549 and H1299 cells; Figure S6: full-sized images of Western blotting (*TRIP13* antibody) following the transfection of siRNAs (si*TRIP13*-1, si*TRIP13*-2) in A549 and H1299 cells; Table S1: reagents used in this study; Table S2: information of tissues by immunostaining.
