# Supplementary material for: Identification of Tumor-Suppressive miR-139-3p-Regulated Genes: TRIP13 as a Therapeutic Target in Lung Adenocarcinoma
Source: Cancers (Basel). 2023 Nov 24;15(23):5571. doi: 10.3390/cancers15235571 (PMC10705761; doi:10.3390/cancers15235571)
Supplement: Supplementary file 1 [file cancers-15-05571-s001.zip › figure Supplement.pptx]

## Slide 1
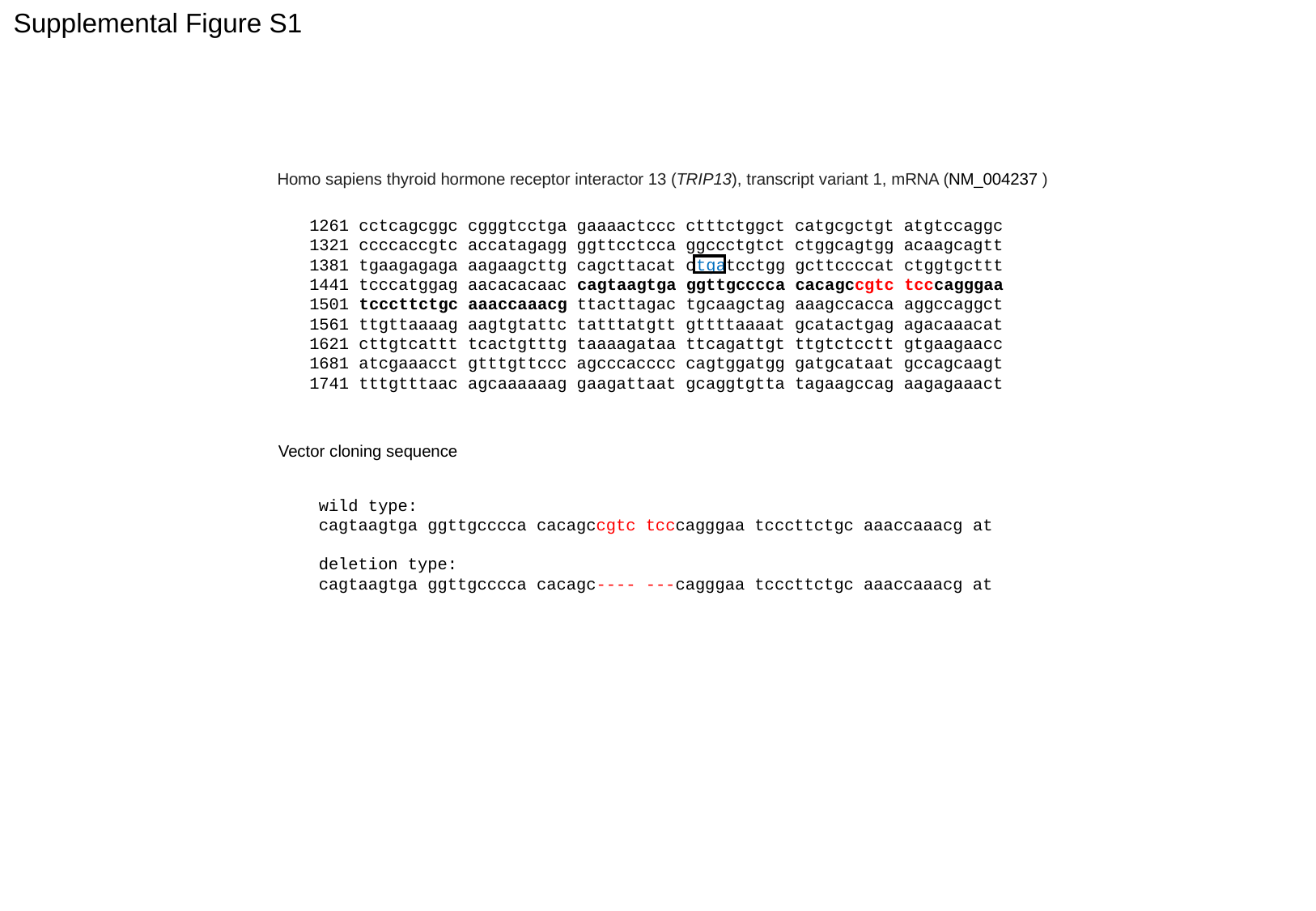

Supplemental Figure S1
Homo sapiens thyroid hormone receptor interactor 13 (TRIP13), transcript variant 1, mRNA (NM_004237 )
 1261 cctcagcggc cgggtcctga gaaaactccc ctttctggct catgcgctgt atgtccaggc
 1321 ccccaccgtc accatagagg ggttcctcca ggccctgtct ctggcagtgg acaagcagtt
 1381 tgaagagaga aagaagcttg cagcttacat ctgatcctgg gcttccccat ctggtgcttt
 1441 tcccatggag aacacacaac cagtaagtga ggttgcccca cacagccgtc tcccagggaa
 1501 tcccttctgc aaaccaaacg ttacttagac tgcaagctag aaagccacca aggccaggct
 1561 ttgttaaaag aagtgtattc tatttatgtt gttttaaaat gcatactgag agacaaacat
 1621 cttgtcattt tcactgtttg taaaagataa ttcagattgt ttgtctcctt gtgaagaacc
 1681 atcgaaacct gtttgttccc agcccacccc cagtggatgg gatgcataat gccagcaagt
 1741 tttgtttaac agcaaaaaag gaagattaat gcaggtgtta tagaagccag aagagaaact
Vector cloning sequence
wild type:
cagtaagtga ggttgcccca cacagccgtc tcccagggaa tcccttctgc aaaccaaacg at
deletion type:
cagtaagtga ggttgcccca cacagc---- ---cagggaa tcccttctgc aaaccaaacg at

## Slide 2
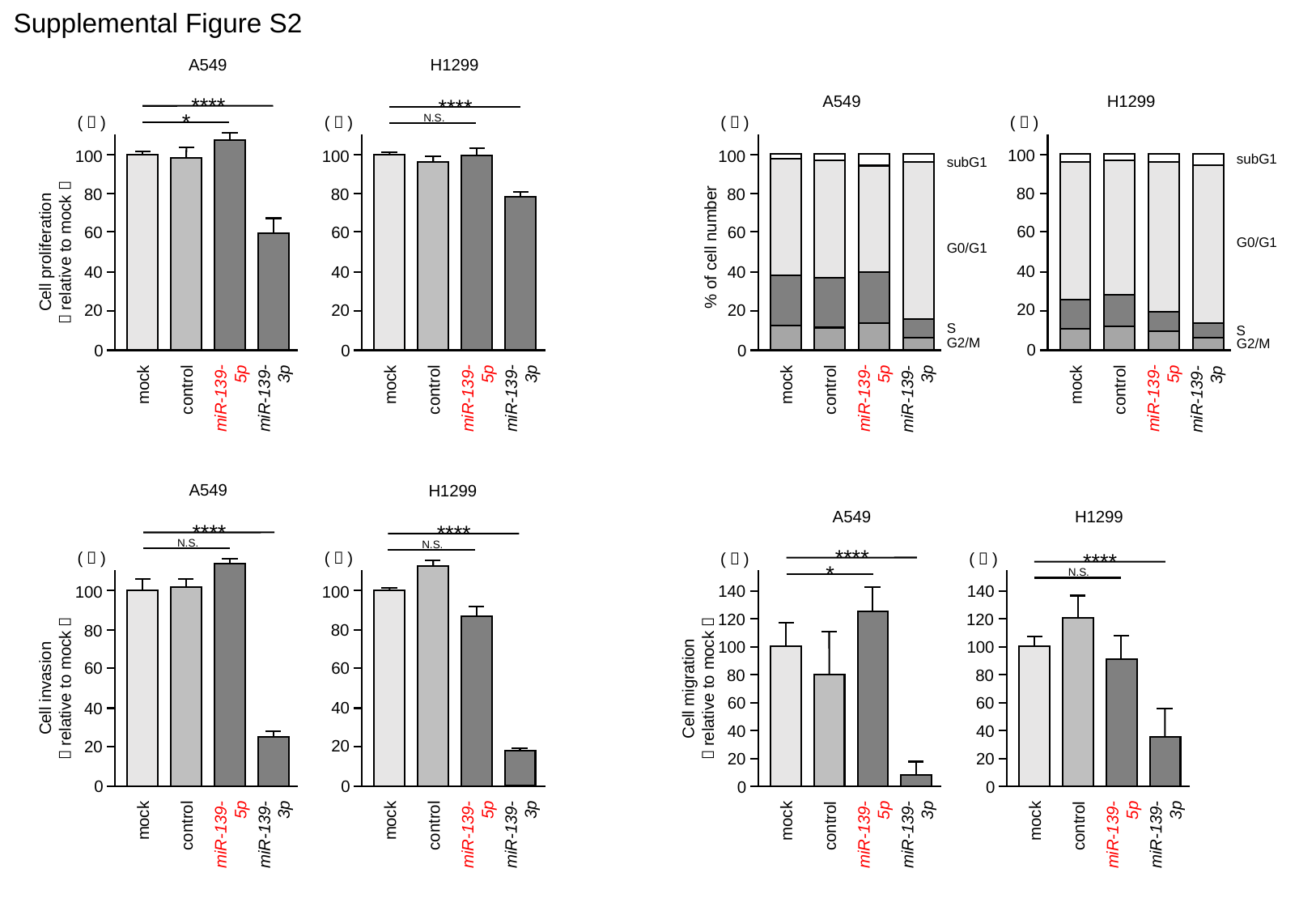

Supplemental Figure S2
A549
****
*
(％)
100
80
60
Cell proliferation
（relative to mock）
40
20
0
mock
control
miR-139-5p
miR-139-3p
H1299
****
N.S.
(％)
100
80
60
40
20
0
mock
control
miR-139-5p
miR-139-3p
A549
(％)
100
subG1
80
60
% of cell number
G0/G1
40
20
S
G2/M
0
mock
control
miR-139-5p
miR-139-3p
H1299
(％)
100
subG1
80
60
G0/G1
40
20
S
G2/M
0
mock
control
miR-139-5p
miR-139-3p
A549
****
N.S.
(％)
100
80
60
Cell invasion
（relative to mock）
40
20
0
miR-139-5p
miR-139-3p
mock
control
H1299
****
N.S.
(％)
100
80
60
40
20
0
mock
control
miR-139-5p
miR-139-3p
H1299
****
(％)
N.S.
140
120
100
80
60
40
20
0
miR-139-3p
mock
control
miR-139-5p
A549
****
(％)
*
140
120
100
80
Cell migration
（relative to mock）
60
40
20
0
miR-139-5p
miR-139-3p
mock
control

## Slide 3
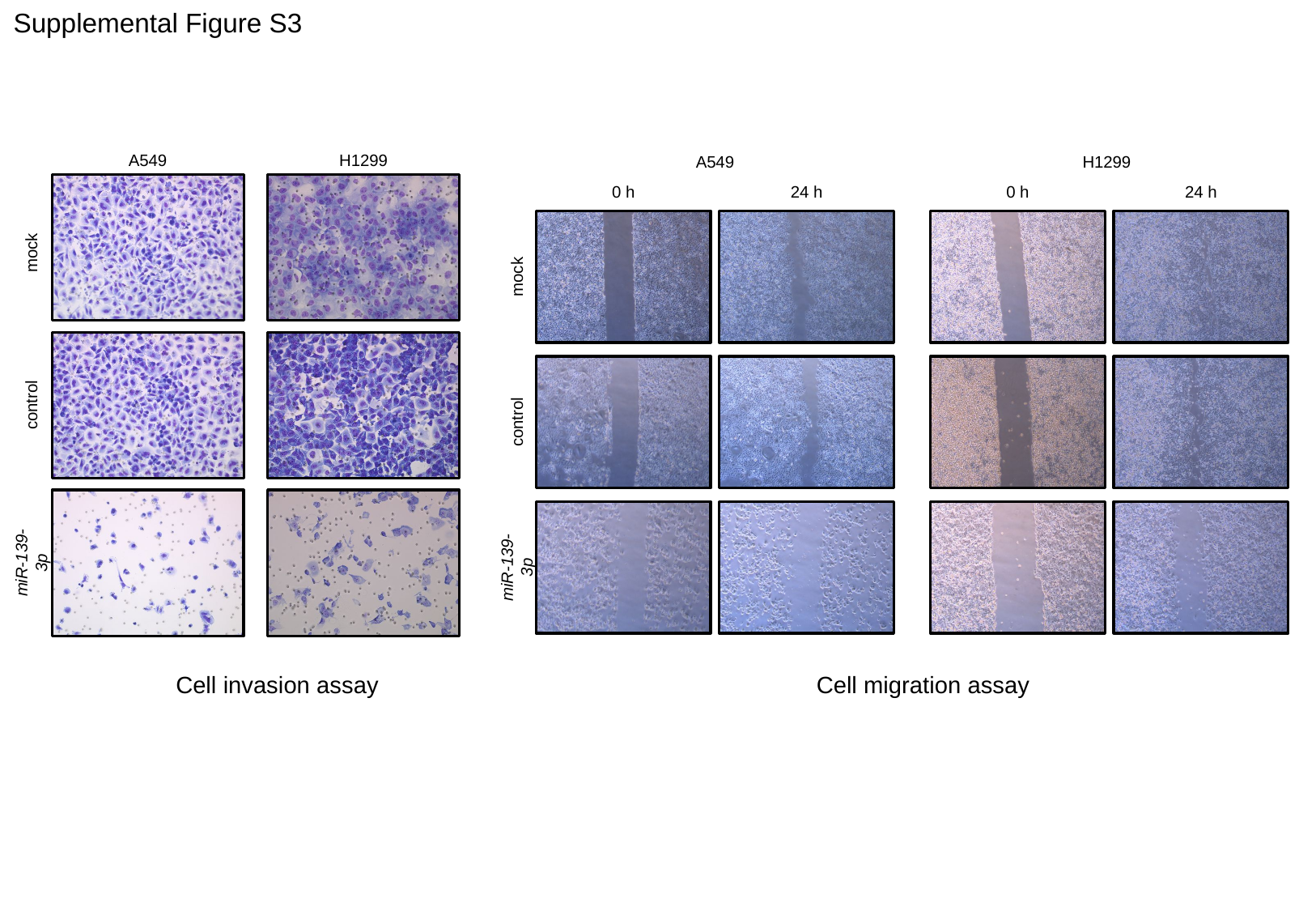

Supplemental Figure S3
A549
H1299
A549
H1299
0 h
24 h
0 h
24 h
mock
mock
control
control
miR-139-3p
miR-139-3p
Cell invasion assay
Cell migration assay

## Slide 4
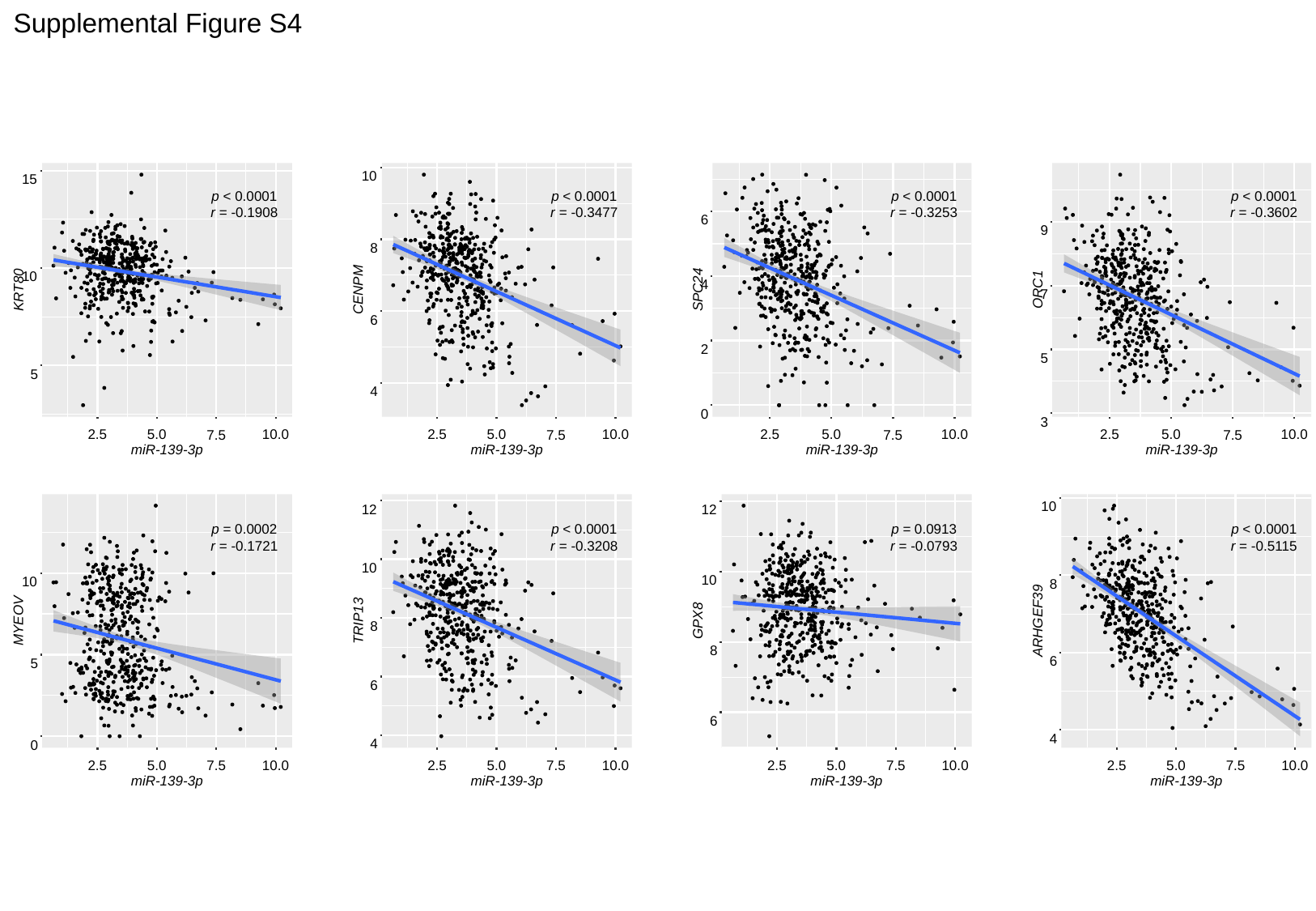

Supplemental Figure S4
15
10
KRT80
5
2.5
5.0
10.0
7.5
miR-139-3p
10
8
CENPM
6
4
2.5
5.0
10.0
7.5
miR-139-3p
6
4
SPC24
2
0
2.5
5.0
10.0
7.5
miR-139-3p
9
7
ORC1
5
3
2.5
5.0
10.0
7.5
miR-139-3p
p < 0.0001
r = -0.1908
p < 0.0001
r = -0.3477
p < 0.0001
r = -0.3253
p < 0.0001
r = -0.3602
10
MYEOV
5
0
2.5
5.0
10.0
7.5
miR-139-3p
12
10
8
TRIP13
6
4
2.5
5.0
10.0
7.5
miR-139-3p
12
10
GPX8
8
6
2.5
5.0
10.0
7.5
miR-139-3p
10
8
ARHGEF39
6
4
2.5
5.0
10.0
7.5
miR-139-3p
p = 0.0002
r = -0.1721
p < 0.0001
r = -0.3208
p = 0.0913
r = -0.0793
p < 0.0001
r = -0.5115

## Slide 5
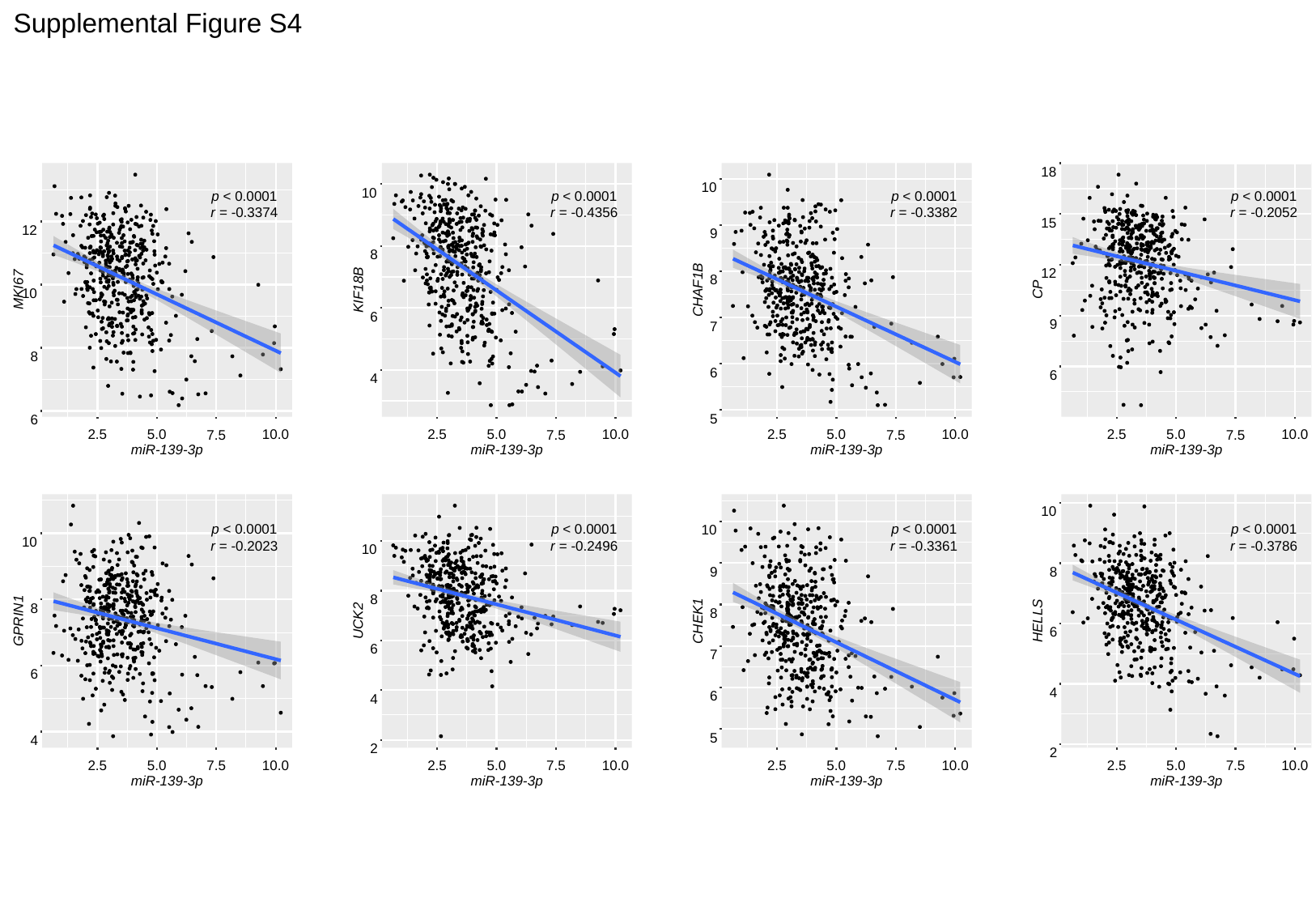

Supplemental Figure S4
12
10
MKI67
8
6
2.5
5.0
10.0
7.5
miR-139-3p
10
8
KIF18B
6
4
2.5
5.0
10.0
7.5
miR-139-3p
10
9
8
CHAF1B
7
6
5
2.5
5.0
10.0
7.5
miR-139-3p
18
15
12
CP
9
6
2.5
5.0
10.0
7.5
miR-139-3p
p < 0.0001
r = -0.3374
p < 0.0001
r = -0.4356
p < 0.0001
r = -0.3382
p < 0.0001
r = -0.2052
10
8
GPRIN1
6
4
2.5
5.0
10.0
7.5
miR-139-3p
10
8
UCK2
6
4
2
2.5
5.0
10.0
7.5
miR-139-3p
10
9
8
CHEK1
7
6
5
2.5
5.0
10.0
7.5
miR-139-3p
10
8
HELLS
6
4
2
2.5
5.0
10.0
7.5
miR-139-3p
p < 0.0001
r = -0.2023
p < 0.0001
r = -0.2496
p < 0.0001
r = -0.3361
p < 0.0001
r = -0.3786

## Slide 6
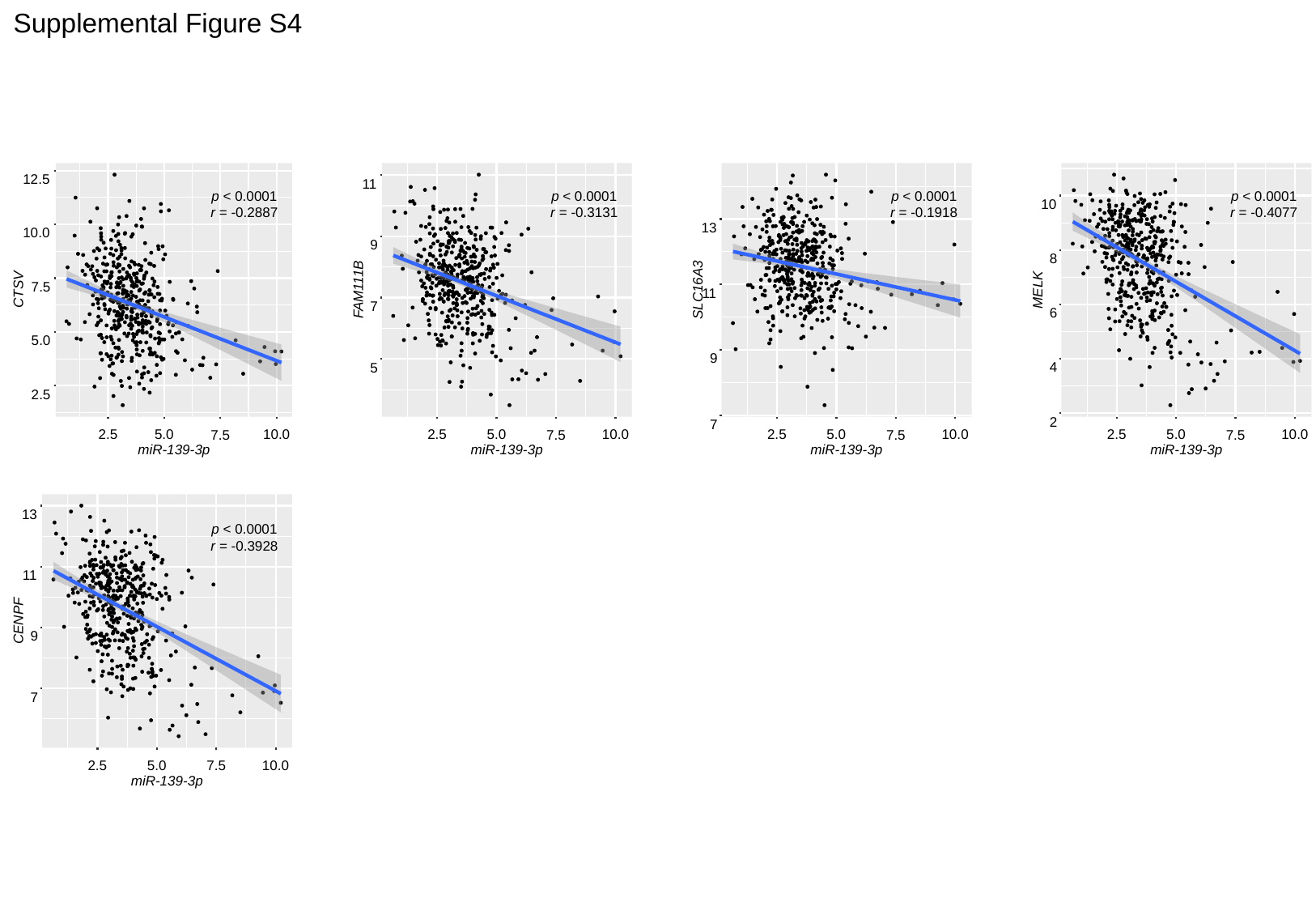

Supplemental Figure S4
12.5
10.0
7.5
CTSV
5.0
2.5
2.5
5.0
10.0
7.5
miR-139-3p
11
9
FAM111B
7
5
2.5
5.0
10.0
7.5
miR-139-3p
13
11
SLC16A3
9
7
2.5
5.0
10.0
7.5
miR-139-3p
10
8
MELK
6
4
2
2.5
5.0
10.0
7.5
miR-139-3p
p < 0.0001
r = -0.2887
p < 0.0001
r = -0.3131
p < 0.0001
r = -0.1918
p < 0.0001
r = -0.4077
13
11
CENPF
9
7
2.5
5.0
10.0
7.5
miR-139-3p
p < 0.0001
r = -0.3928

## Slide 7
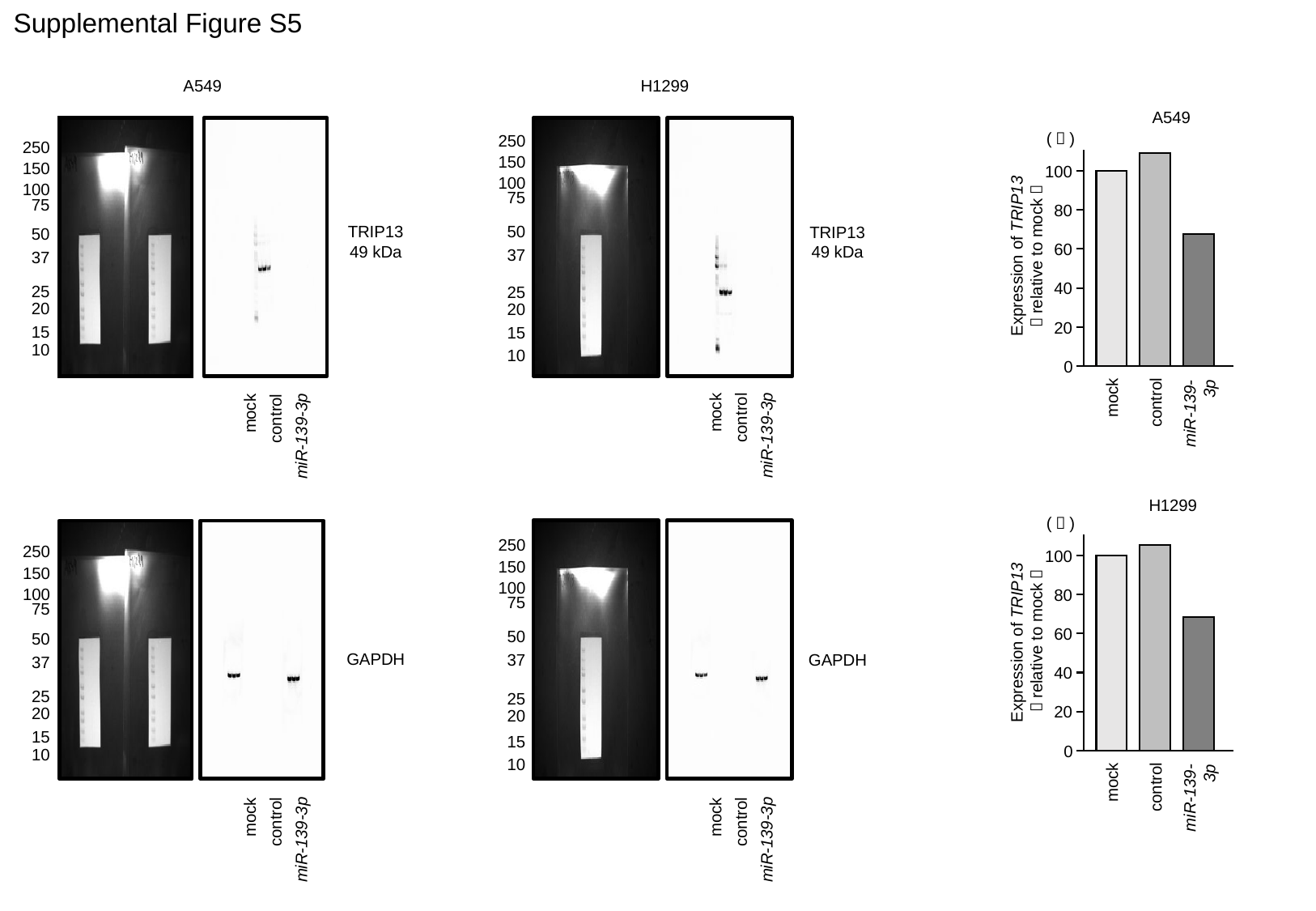

Supplemental Figure S5
A549
H1299
A549
(％)
250
250
150
150
100
100
100
75
75
80
50
TRIP13
49 kDa
TRIP13
49 kDa
50
Expression of TRIP13
（relative to mock）
60
37
37
40
25
25
20
20
20
15
15
10
10
0
mock
control
miR-139-3p
mock
control
miR-139-3p
mock
control
miR-139-3p
H1299
(％)
250
250
100
150
150
100
100
80
75
75
Expression of TRIP13（relative to mock）
60
50
50
GAPDH
37
GAPDH
37
40
25
25
20
20
20
15
15
0
10
10
mock
control
miR-139-3p
mock
control
miR-139-3p
mock
control
miR-139-3p

## Slide 8
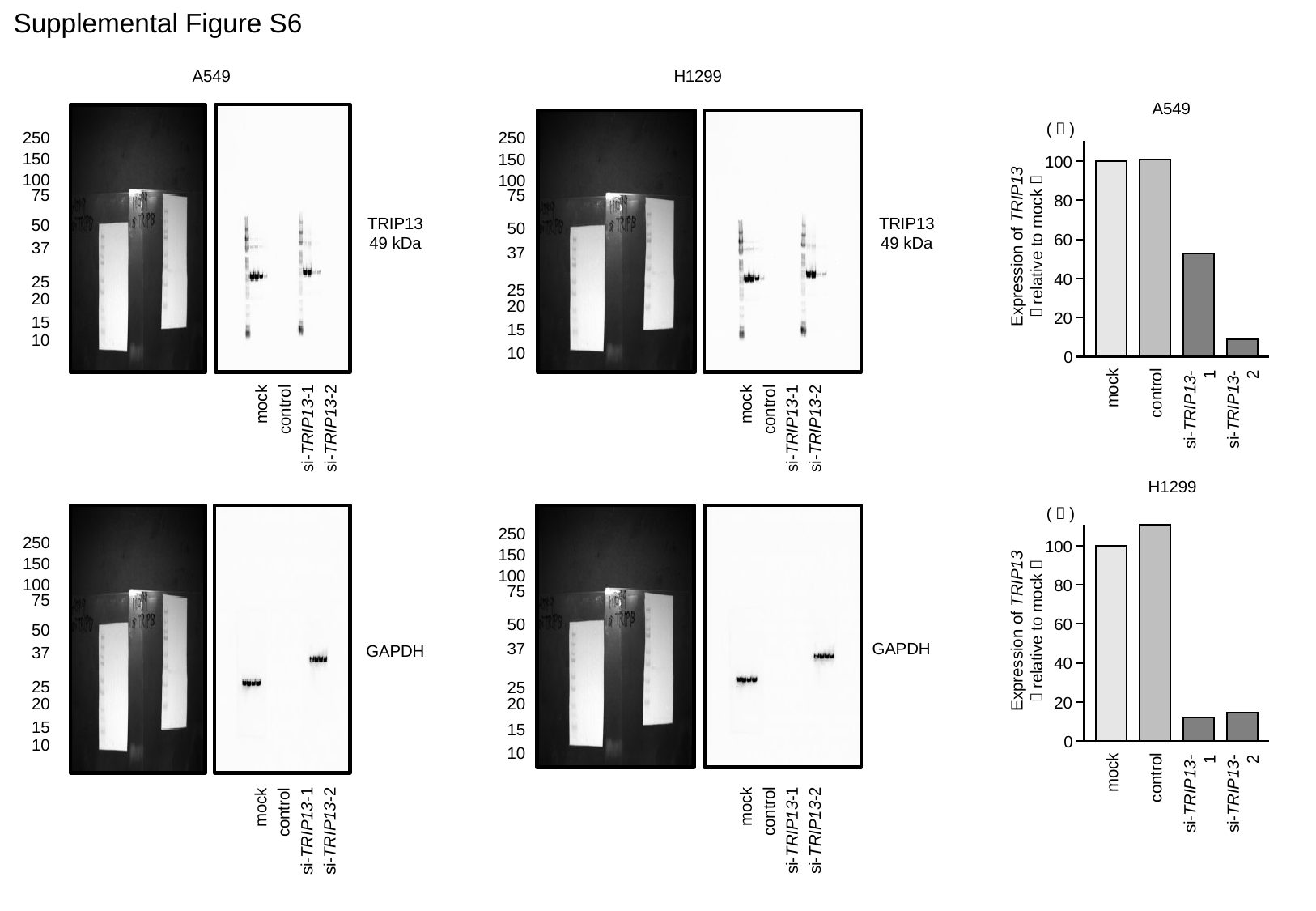

Supplemental Figure S6
A549
H1299
A549
(％)
250
250
150
150
100
100
100
75
75
80
TRIP13
49 kDa
TRIP13
49 kDa
50
50
Expression of TRIP13
（relative to mock）
60
37
37
40
25
25
20
20
20
15
15
10
10
0
mock
control
si-TRIP13-2
si-TRIP13-1
mock
control
si-TRIP13-1
si-TRIP13-2
mock
control
si-TRIP13-1
si-TRIP13-2
H1299
(％)
250
250
100
150
150
100
100
80
75
75
Expression of TRIP13
（relative to mock）
50
60
50
37
GAPDH
GAPDH
37
40
25
25
20
20
20
15
15
0
10
10
mock
control
si-TRIP13-2
si-TRIP13-1
mock
control
si-TRIP13-1
si-TRIP13-2
mock
control
si-TRIP13-1
si-TRIP13-2
